# Patterns and Risk Factors of Helminthiasis and Anemia in a Rural and a Peri-urban Community in Zanzibar, in the Context of Helminth Control Programs

**DOI:** 10.1371/journal.pntd.0000681

**Published:** 2010-05-11

**Authors:** Stefanie Knopp, Khalfan A. Mohammed, J. Russell Stothard, I. Simba Khamis, David Rollinson, Hanspeter Marti, Jürg Utzinger

**Affiliations:** 1 Department of Epidemiology and Public Health, Swiss Tropical and Public Health Institute, Basel, Switzerland; 2 Helminth Control Laboratory Unguja, Ministry of Health and Social Welfare, Zanzibar, Tanzania; 3 Wolfson Wellcome Biomedical Laboratories, Department of Zoology, Natural History Museum, London, United Kingdom; 4 Department of Medical and Diagnostic Services, Swiss Tropical and Public Health Institute, Basel, Switzerland; George Washington University, United States of America

## Abstract

**Background:**

The control of helminth infections and prevention of anemia in developing countries are of considerable public health importance. The purpose of this study was to determine patterns and risk factors of helminth infections and anemia in a rural and a peri-urban community of Zanzibar, Tanzania, in the context of national helminth control programs.

**Methodology/Principal Findings:**

We carried out a community-based cross-sectional study in 454 individuals by examining at least two stool samples with different methods for soil-transmitted helminths (*Ascaris lumbricoides*, hookworm, *Strongyloides stercoralis*, and *Trichuris trichiura*) and one urine sample for *Schistosoma haematobium*. Finger-prick blood was taken to estimate anemia levels and to detect antibody reactions against ascariasis, strongyloidiasis and schistosomiasis, using an enzyme-linked immunosorbent assay (ELISA) approach. Parasitological methods determined a helminth prevalence of 73.7% in the rural, and 48.9% in the peri-urban setting. Most helminth infections were of light intensity with school-aged children showing the highest intensities. Multiple helminth species infections were pervasive in rural dwellers regardless of age. More than half of the participants were anemic, with a particularly high prevalence in the peri-urban setting (64.7%). Risk factors for helminth infections were age, sex, consumption of raw vegetables or salad, recent travel history, and socio-economic status.

**Conclusion/Significance:**

After several years of chemotherapy-based morbidity control efforts in Zanzibar, helminth prevalences are still high and anemia is common, but helminth infection intensities are low. Hence, chemotherapy should be continued, and complemented with improved access to clean water, adequate sanitation, and health education, along with poverty alleviation measures for a more enduring impact.

## Author Summary

In many parts of the developing world, parasitic worms and anemia are of considerable public health and economic importance. We studied the patterns and risk factors of parasitic worm infections in a rural and a peri-urban community on Zanzibar Island, Tanzania, in the context of national deworming programs. We invited 658 individuals aged between 5 and 100 years and examined their stool and urine for the presence of parasitic worm eggs. Additionally, we obtained a finger-prick blood sample to estimate the level of anemia and to assess for specific immune reactions against parasitic worm infections. We found that, despite large-scale deworming efforts in Zanzibar over the past 15 years, three-quarter of the rural participants and half of the peri-urban residents were infected with parasitic worms. Every second participant was anemic. Risk factors for a parasitic worm infection were age, sex, consumption of raw vegetables or salad, recent travel history, and socio-economic status. For a sustainable control of parasitic worm infections and prevention of anemia, access to safe and efficacious drugs, complemented with health education and improvements in water supply and adequate sanitation are necessary.

## Introduction

Soil-transmitted helminthiasis and schistosomiasis affect hundreds of millions of people and the global burden due to these parasitic worms might exceed 40 million disability-adjusted life years (DALYs) lost annually [1]–[3]. Chronic infections can result in negative birth outcomes, delayed physical and cognitive development during childhood, and reduced agricultural productivity among adults [4], [5]. There is growing evidence that, besides malaria and nutritional deficiencies, heavy helminth infections as well as multiple helminth species infections of light intensities, are associated with anemia [6]–[8].

The global strategy for the control of soil-transmitted helminthiasis and schistosomiasis is morbidity control using single-dose orally-active anthelmintic drugs. In 2001, at the 54^th^ World Health Assembly (WHA), member states were urged to achieve a minimum target of regular deworming of at least 75% and up to 100% of school-aged children and other groups at risk of morbidity by 2010 [9].

In Zanzibar, infections with soil-transmitted helminths (*Ascaris lumbricoides*, hookworm, and *Trichuris trichiura*) are highly endemic [10]–[12]. *Strongyloides stercoralis* infections also occur, but few epidemiological investigations have focused on this helminth [11], [13]. With regard to schistosomiasis, *Schistosoma haematobium* is the only species endemic in Zanzibar. Its distribution is focal, linked to the distribution of the intermediate host snail [12], [14]. Due to excessively high helminth prevalences (>90%) in school-aged children in Zanzibar, large-scale school-based administration of anthelmintic drugs (mebendazole or albendazole) was initiated in the mid-1990s [15], and contingent upon drug donations deworming has been carried out annually. Of note, in 2006, Zanzibar achieved the minimum target of regular administration of anthelmintic drugs to at least 75% of all school-aged children [16]. In addition, within the global program to eliminate lymphatic filariasis (GPELF), ivermectin plus albendazole were distributed to the entire at-risk population (excluding children younger than 5 years and pregnant women) in Zanzibar from 2001 to 2006, and a mean annual coverage rate of 80% was reached [17]–[19]. Importantly, ivermectin is efficacious against *S. stercoralis* [13] and albendazole against common soil-transmitted helminths [20].

Large-scale deworming programs in Zanzibar have reduced helminth-related morbidity and are likely to have lowered overall transmission [17], [21]. Hence, as original programmatic targets are being met, it is interesting to study the patterns and risk factors of helminth infections in contemporary times. The aim of our cross-sectional study was to assess the current prevalence and intensity of helminth infections and to determine anemia levels in different age groups in a rural and a peri-urban community in Zanzibar, in the context of helminth control programs. We used standardized, quality-controlled parasitological and serological tests, administered a questionnaire to investigate behavioral, demographic, and socio-economic risk factors for helminth infection and anemia, and determined self-reported morbidity signs that might be associated with helminth infections. Combined, this information will provide an important insight into the impact of ongoing interventions and hopefully evidence-based realignment of disease-control priorities.

## Materials and Methods

### Ethics statement

The study protocol was approved by the institutional research commission of the Swiss Tropical and Public Health Institute (Basel, Switzerland). Ethical clearance was obtained from the Ethics Committee of the Ministry of Health and Social Welfare (MoHSW) in Zanzibar (application number 16). The shehas (community leaders/heads) and sub-shehas of each shehia (administrative area) were informed about the purpose and procedures of the study. The inhabitants of the shehia were subsequently informed by the shehas. All adult participants and the parents/legal guardians on behalf of their children (aged 5–16 years) signed a written informed consent sheet. All participants were free to withdraw from the study at any time without further obligation. At the end of the study all participants were invited to learn about their parasitological results and were treated with albendazole (single 400 mg oral dose) if infected with *A. lumbricoides*, *T. trichiura* and/or hookworm, with ivermectin (single 200 µg/kg oral dose) if infected with *S. stercoralis*, and with praziquantel (single 40 mg/kg oral dose) if infected with *S. haematobium*.

### Study setting

The Zanzibar archipelago consists of the two main islands of Unguja and Pemba with ∼1 million inhabitants according to the population census in 2002. Fishing and farming are the most important economic activities. Islam is the predominant religion.

Our community-based cross-sectional study was carried out in two shehias of Unguja, in collaboration with the Helminth Control Laboratory Unguja (HCLU) of the MoHSW in June and July 2008. Bandamaji is a rural shehia, located in District North A, ∼40 km from Zanzibar Town. According to the 2002 census its population consisted of 993 inhabitants and the annual growth rate in District North A is 2.4% [22]. Dole is a peri-urban shehia in District West, located ∼10 km north-west from Zanzibar Town. There were 2,496 inhabitants in 2002. The annual growth rate of District West is 9.2% [23]. Both sites received single annual treatments of albendazole and ivermectin in the frame of the GPELF from 2001–2006, and albendazole or mebendazole plus praziquantel via the school-based deworming program till 2006. Of note, the school in Bandamaji was only opened in 2005.

### Study participants

According to guidelines put forth by the World Health Organization (WHO), a sample size of 250 complying individuals in a geographically distinct community is needed to assess the prevalence and intensity of soil-transmitted helminth infections [24], [25]. Allowing for drop-out and non-compliance of 20–30%, we aimed at enrolling approximately 330 individuals per study setting, with a quarter being school-aged children (5–15 years) and the remaining three-quarter being adolescents and adults (>15 years). All individuals from Bandamaji and Dole aged 5 years and above were eligible for the study.

The shehas were asked to invite ∼300 adult community members to attend an information meeting and to bring along their children. After the meeting, adolescents and adults who participated in the meeting were invited to sign an informed consent form. Additionally, children on the spot and their parents/legal guardians were asked for the children's age and whether they were interested to participate in the study. Subsequently, the first ∼50 girls and the first ∼50 boys aged 5–15 years, who lined up to receive a consent form and were accompanied by their parents or legal guardians who signed the form were enrolled in the study.

### Field procedures

The shehas of both communities were informed about the purpose of the study. After obtaining their oral consent the shehas participated actively in deciding how, when and where the specific parts of the study (e.g., information of participants, collection of stool, urine, and blood, and day for questionnaire survey) should take place. After a further meeting where the aims of the study were explained in lay terms in the local language Kiswahili to ∼300 invited adult community members and participating children, written informed consent was obtained and three stool containers labeled with unique identifiers and the intended collection day were distributed. Our goal was to obtain three stool samples over consecutive days. Stool samples were collected between 08:00 and 10:00 hours by fieldworkers at a public spot and transferred to the HCLU in Zanzibar Town.

On Friday, when people tend to stay near their village for prayer, additional field procedures were carried out. Participants were interviewed with a pre-tested questionnaire about risk factors and morbidity signs that might be related to helminth infections and anemia, and about household characteristics and asset ownership [26]. Subsequently, finger-prick blood was collected from each participant and the hemoglobin (Hb) level was determined with a portable Hemocue device (HemoCue Hb 201+; Sheffield, United Kingdom). Finger-prick blood samples were collected in small tubes (BD Microtainer, Ref.: 365967) and put on ice after clotting. Finally, participants were invited to submit a urine sample, which was collected between 10:00 and 14:00 hours.

### Laboratory procedures

#### Parasitological examinations

Stool and urine samples were collected to screen for helminth eggs using standardized, quality-controlled parasitological tests. All stool samples were examined at HCLU using the Kato-Katz (K-K) [27], the Baermann (BM) [28], and the Koga agar plate (KAP) method [29]. Details of these methods have been provided elsewhere [30]. In brief, 41.7 mg K-K thick smears were read under a microscope within 20–40 min to avoid rapid over-clearance of hookworm eggs in glycerol [31]. The number of *A. lumbricoides*, hookworm, and *T. trichiura* eggs were counted and recorded separately. Regarding the BM method, ∼5 g of stool was put on medical gauze within a glass funnel that was filled with water and exposed to an electric light source from below. Phototactic larvae were collected after 2 hours of exposure and visualized on microscope slides. The number of *S. stercoralis* larvae was recorded for each participant. Finally, regarding KAP, ∼2 g of stool was exposed in the middle of an agar plate, and the plates were incubated for 48 hours in a humid chamber before visual examination for traces of *S. stercoralis* and/or hookworm larvae. Subsequently, the plates were washed with 10% formaldehyde and the sediment was qualitatively examined for *S. stercoralis* and hookworm larvae under a microscope.

For quality control, a senior laboratory technician re-examined a random sample of 10% of the K-K thick smears daily. If false-negatives were identified or if differences in egg counts were observed that were judged unacceptable by the study coordinator (S.K.), the respective microscopist was advised to read more carefully the next day and original results were replaced by the results of the senior technician. If there were differences judged unacceptable in more than 20% of slides subjected to quality control, all slides of the day were re-examined.

Eggs of *S. haematobium* were counted under a microscope after filtering 10 ml of vigorously shaken urine with a syringe using a 12 µm polycarbonate millipore filter (Millipore; Billerica, MA, USA) [32].

#### Serological examinations

Serum was collected to screen for antibodies against *Ascaris*, *Strongyloides*, and *Schistosoma* antigens using commercially available ELISA kits (Microwell Serum ELISA, IVD Research Inc.; Carlsbad, CA, United States of America). At HCLU, the clotted finger-prick blood samples were centrifuged at 6,000 g for 10 min in a benchtop microcentrifuge (Eppendorf Mini Spin; Hamburg, Germany). The sera were transferred into labeled Eppendorf tubes and stored at −20°C pending further analyses. For ELISA, sera were prepared following the manufacturer's manual. In brief, freshly thawed sera were diluted 1∶100, 1∶64 and 1∶40 in dilution buffers for *A. lumbricoides*, *S. stercoralis*, and *S. haematobium*-specific ELISAs, respectively. Next, 100 µl of each serum dilution was transferred into one well of three microtiter plates coated by the manufacturer with *Ascaris*, *Strongyloides*, and *Schistosoma* antigens, respectively. At least one positive and one negative control provided by the manufacturer were included in each test. After steps of incubation, washing, adding the enzyme conjugate (protein A-peroxidase) and the chromogen tetramethylbenzidine substrate and finally stopping the reaction with 1 M phosphoric acid, the plates were read at an optical density (OD) of 450 nm using an ELISA reader (Multiskan Bichromatic, version 1.06 P; Vienna, VA, United Sates of America) according to the manufacturer's instructions.

### Questionnaire survey

Participants were interviewed in Kiswahili with a pre-tested questionnaire by trained field enumerators of HCLU. The questionnaire included demographic and a series of housing characteristics (i.e., building type of walls, floor, and roof) and asset ownership (i.e., mobile phone, radio, black and white television (TV), color TV, satellite dish, video compact disc player, fan, refrigerator, bicycle, motorbike, car, stove type (electric, coal, wood), soap, access to the power grid, and animals (cattle, cow, goat, sheep, and donkey)). Risk factors for a helminth infection were determined via source of drinking water used (i.e., tap, shallow well, deep well, spring, and river), presence and type of toilet at home (no toilet–using “the bush”, latrine, or flush toilet), hand washing behavior (whether or not hands are washed with or without soap before eating and after defecation), consumption of raw vegetables or salad, consumption of unpeeled fruits, consumption of soil, always wearing shoes, sleeping under a bed net, traveling within the last two weeks, and ownership of a dog or a cat. Finally, using a recall period of two weeks, the questionnaire included 12 morbidity signs (i.e., fever, fatigue, stomach ache, diarrhea, blood in stool, blood in urine, pain when urinating, vomiting, cough, blood in sputum, itching, and headache) and six diseases (i.e., malaria, soil-transmitted helminthiasis, schistosomiasis, skin disease, eye disease, and cold).

### Data management and statistical analysis

Parasitological and serological data were entered twice in Microsoft Excel version 10.0 (2002 Microsoft Corporation). Questionnaire data were entered twice in EpiInfo version 3.5.1 (Centers for Disease Control and Prevention; Atlanta, GA, United States of America). Double-entered datasets were compared using EpiInfo and discrepancies removed against the original records. Data were analyzed using STATA version 9.2 (StataCorp.; College Station, TX, United States of America) and R version 2.10.1 (R Development Core Team; Vienna, Austria). Only individuals who submitted at least two stool samples were included in the final analyses.

Age was stratified into four groups, (i) 5–11 years, (ii) 12–14 years, (iii) 15–59 years, and (iv) ≥60 years, as suggested by WHO [33], [34]. For each individual, the arithmetic mean of the helminth species-specific egg counts from the K-K thick smears was calculated and multiplied by a factor 24 to obtain a standardized measure of infection intensity, expressed as eggs per gram of stool (EPG). Infection intensities were classified into light, moderate, and heavy, according to thresholds put forward by WHO [9]. The lower limits of moderate and heavy infections were 5,000 and 50,000 EPG for *A. lumbricoides*, 1,000 and 10,000 EPG for *T. trichiura*, and 2,000 and 4,000 EPG for hookworm, respectively. *S. haematobium* egg counts were classified into light (1–49 eggs/10 ml of urine) and heavy (≥50 eggs/10 ml of urine) [9].

Differences in the median EPG of the four age groups were determined using the Kruskal-Wallis test. Pair-wise comparisons between the median EPG of two age groups were adjusted for multiple testing as suggested by Siegel and Castellan [35]. Statistical significance was given at a p-value of 0.05. Hb thresholds used to define anemia were 115 g/l for children of both sexes aged 5–11 years, 120 g/l for children of both sexes aged 12–14 years, 120 g/l for women aged ≥15 years (non-pregnant) and 130 g/l for men aged ≥15 years, following WHO thresholds [33]. Anemia was classified into ‘moderate to severe anemia’ and ‘heavy anemia’ when Hb values were <90 g/l and <70 g/l, respectively [36].

Antibody reactions were regarded as positive when the absorbance readings were >0.1 OD units for *A. lumbricoides*, >0.2 OD units for *S. stercoralis*, and ≥0.2 OD units for *S. haematobium*, following the manufacturer's handbook. Sensitivity (i.e., proportion of true-positives identified as positive) and specificity (i.e., proportion of true-negatives identified as negative) of the ELISA tests were determined considering the pooled results of the respective ELISA and at least two K-K this smear readings (for *A. lumbricoides*), one urine filtration (for *S. haematobium*), or at least two KAP and/or BM (for *S. stercoralis*) as diagnostic ‘gold’ standard. Hence, a person was considered positive for a particular parasite if at least one diagnostic test revealed a positive result.

The socio-economic status was determined according to a wealth index, calculated on the basis of housing characteristics and asset ownership. Using principal component analysis (PCA), based on the sum of household and asset characteristic scores, all interviewed participants were grouped into wealth quintiles: (i) most poor, (ii) very poor, (iii) poor, (iv) less poor, and (v) least poor [37], [38]. Multivariable logistic regression was used for estimating odds ratios (ORs), including 95% confidence intervals (CIs), to determine risk factors for helminth infections and anemia, and to determine associations between helminth infections and anemia and self-reported morbidity signs and diseases, as assessed by the questionnaire. Outcomes were defined as specific helminth infection, determined by any parasitological method in at least one stool or urine sample, or the presence of anemia, or any self-reported morbidity sign or disease. Candidate explanatory variables for the multivariable logistic regression were those which were reasonable and present in at least 5% of the interviewed participants in each community. A backward stepwise multivariable logistic regression, allowing for possible clustering within houses and stratified by community, and removing non-predicting covariates up to a significance level of 0.2 was performed using the sandwich estimator robust cluster option in STATA. The remaining variables were included into the final models and the Wald test was used to determine whether each independent variable was significantly related to the outcome variable.

## Results

### Study cohort and compliance

**Figure 1:**
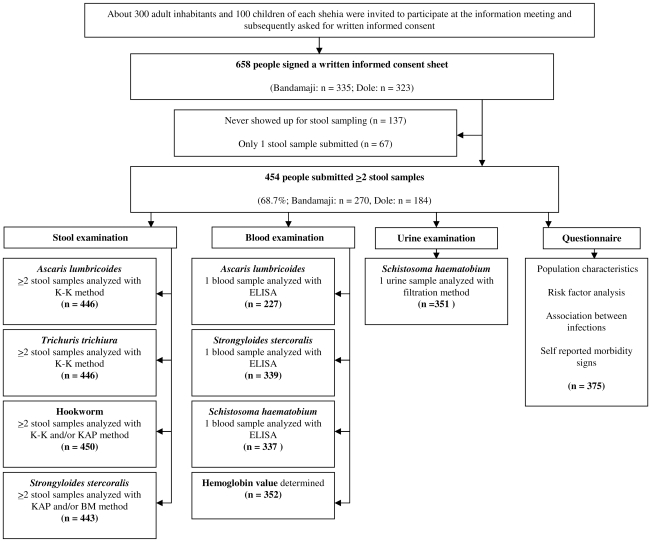
Study participation and compliance. Diagram detailing the study participation and compliance of community members from rural Bandamaji and peri-urban Dole, Zanzibar, in June/July 2008. All individuals providing at least two stool samples were included in the final analyses. K-K: Kato-Katz method, KAP: Koga agar plate method, BM: Baermann method.

From 658 individuals who registered for the study and signed a written informed consent sheet, 137 (20.8%) never submitted any stool sample. A single stool sample was provided by 67 (10.2%) participants. The remaining 454 individuals (69.0%) submitted two or three stool samples, and hence were included for further analyses (Figure 1). Among them, 294 were female (64.8%) and 160 were male (35.2%). There were 270 people from Bandamaji (59.5%) and 184 from Dole (40.5%). The age groups of 5–11, 12–14, 15–59 and ≥60 years consisted of 106, 74, 231 and 43 individuals, respectively. The median age was 19.5 years.

Due to insufficient quantities of feces and the priority for the sequence of tests employed, 446 (98.2%), 437 (96.3%) and 411 (90.5%) individuals had at least two stool samples examined with the K-K, KAP, and BM method, respectively. Since for hookworm diagnosis the results of both K-K and KAP, and for *S. stercoralis* diagnosis the results of both KAP and BM were combined, the respective analyses included 450 (99.1%) and 443 (97.6%) individuals. Urine samples for *S. haematobium* examination were available from 351 individuals (77.3%). Finger-prick blood for estimating Hb levels was available from 352 participants (77.5%). Antibody reactions against *S. haematobium*, *S. stercoralis*, and *A. lumbricoides*, were tested using ELISA from 339 (74.7%), 337 (74.2%), and 227 (50.0%) participants, respectively The questionnaire was completed by 375 out of the 454 individuals who submitted at least two stool samples (82.6%).

### Population profile

Key population characteristics as determined by the questionnaire survey, stratified by study settings (rural Bandamaji and peri-urban Dole), are presented in Table 1. Both populations differed significantly as for place of birth, religion, profession, educational attainment, and socio-economic status. In summary, most of the rural dwellers were born in Unguja and most adolescents/adults (≥16 years) resided in Bandamaji for more than 10 years. They were all Muslim and farming was their primary occupation. Only 9.8% of the participants belonged to the least poor wealth quintile.

**Table 1:** Population characteristics, according to questionnaire survey, stratified by study setting (rural Bandamaji: n = 236, peri-urban Dole: n = 139) in Zanzibar, June/July 2008.

Population characteristics	Bandamaji		Dole		Difference	
	n	%	n	%	χ^2^	P-value
**Place of birth**						
Unguja	233	98.7	87	62.6		
Pemba	1	0.4	8	5.8		
Tanzania mainland	2	0.9	43	30.9		
Not known	0	0	1	0.7	91.4	<0.001
**Residency in village** a						
<2 years	2	1.4	5	6.4		
2–5 years	7	5.0	5	6.4		
6–10 years	10	7.1	7	9.0		
>10 years	119	84.4	60	76.9		
Not applicable	3	2.1	1	1.3	4.9	0.300
**Religion**						
Islam	236	100	116	83.5		
Christian	0	0	21	15.1		
Other	0	0	2	1.4	41.6	<0.001
**Occupation**						
Preschool child	6	2.5	14	10.1		
School child	98	41.5	51	36.7		
Farmer	110	46.6	49	35.3		
Trader	2	0.9	5	3.6		
Teacher/civil servant	4	1.7	6	4.3		
Other	1	0.4	5	3.6		
Not known	15	6.4	9	6.5	23.8	0.001
**Educational attainment** b						
Illiterate	65	49.2	23	31.1		
Pre school	1	0.8	1	1.4		
Primary school	51	38.6	26	35.1		
Secondary school	13	9.9	20	27.0		
Middle school/apprenticing	2	1.5	3	4.1		
Junior college or university	0	0	1	1.4	15.8	0.008
**Socio-economic status**						
Most poor	56	23.7	19	13.7		
Very poor	62	26.3	13	9.4		
Poor	50	21.2	25	18.0		
Less poor	45	19.1	31	22.3		
Least poor	23	9.8	51	36.7	50.0	<0.001

a Considered only individuals aged ≥16 years (Bandamaji: n = 141, Dole: n = 78).

b Considered only individuals who were not preschool or school children, according to occupation (Bandamaji: n = 132, Dole: n = 74).

In contrast, more than a third of the peri-urban dwellers were born outside Unguja and almost a quarter of the interviewed adolescents/adults had lived in Dole for less than 10 years. Islam is the predominant religion, but there were also Christians (15.1%). The range of occupations in Dole was broader than in Bandamaji, including a higher percentage of traders, teachers, and civil servants. More than a third of the peri-urban inhabitants belonged to the least poor wealth quintile.

### Helminth infections and anemia, stratified by study setting

The overall prevalence of infection with any helminth species, according to different tests examined under a microscope was 73.7% in Bandamaji and 48.9% in Dole. In Bandamaji, the prevalence of *A. lumbricoides*, *T. trichiura*, hookworm, *S. stercoralis*, and *S. haematobium* was 49.4%, 48.7%, 31.1%, 10.3%, and 5.4%, respectively. In Dole, hookworm was the predominant species (32.2%). Infections with *A. lumbricoides* were rare (3.4%), whereas prevalences of 16.8%, 12.7%, and 11.7% were found for *T. trichiura*, *S. stercoralis*, and *S. haematobium*.

Among the infected individuals, 87.0% (120/138) and 13.0% (18/138) had a light and moderate infection with *A. lumbricoides*, 98.7% (158/160) and 1.3% (2/160) had a light and moderate infection with *T. trichiura* and, with the exception of one moderate hookworm infection, all others 99.1% (105/106) were of light intensity. No heavy infections were found for any soil-transmitted helminth. All moderate infection intensities with both *A. lumbricoides* and *T. trichiura* were observed in Bandamaji, whereas the moderate hookworm infection was found in Dole. Among the 27 *S. haematobium* infections, 22.2% (four individuals from Dole and two from Bandamaji) were heavily infected.

The prevalence of anemia was 64.7% in Dole and 50.9% in Bandamaji, with 8.1% (16/198) individuals showing moderate-to-severe anemia, and 2.5% (5/198) individuals being severely anemic.

### Helminth infections and anemia, stratified by age group

**Figure 2:**
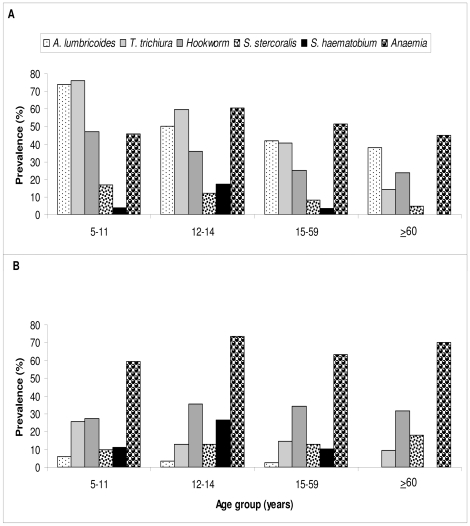
Prevalence of soil-transmitted helminths, *S. haematobium* and anemia in rural Bandamaji and peri-urban Dole, Zanzibar, in June/July 2008. Bar chart indicating the prevalence of soil-transmitted helminths, *S. haematobium* and anemia in four age groups of (A) rural Bandamaji, and (B) peri-urban Dole. (A) age group 5–11 years: n = 55; age group 12–14 years: n = 42; age group 15–59 years: n = 152; age group ≥60 years: n = 21. (B) age group 5–11 years: n = 51; age group 12–14 years: n = 32; age group 15–59 years: n = 79; age-group ≥60 years: n = 22.

The species-specific prevalence of helminth infections in each of the four age groups in Bandamaji and Dole is presented in Figure 2. In both settings, the prevalence of *A. lumbricoides* and *T. trichiura* decreased with age. Whilst a decrease with age was also observed for hookworm and *S. stercoralis* in Bandamaji, the age-prevalence curve for these two helminths was relatively stable in Dole. Regardless of the study setting, *S. haematobium* infections were most prevalent in the 12–14-year-old age group (17.1% in Bandamaji and 26.3% in Dole). No *S. haematobium* infections were found in the elderly (≥60 years). Finally, anemia peaked in the age group 12–14 years (60.6% in Bandamaji, 73.7% in Dole).

**Figure 3:**
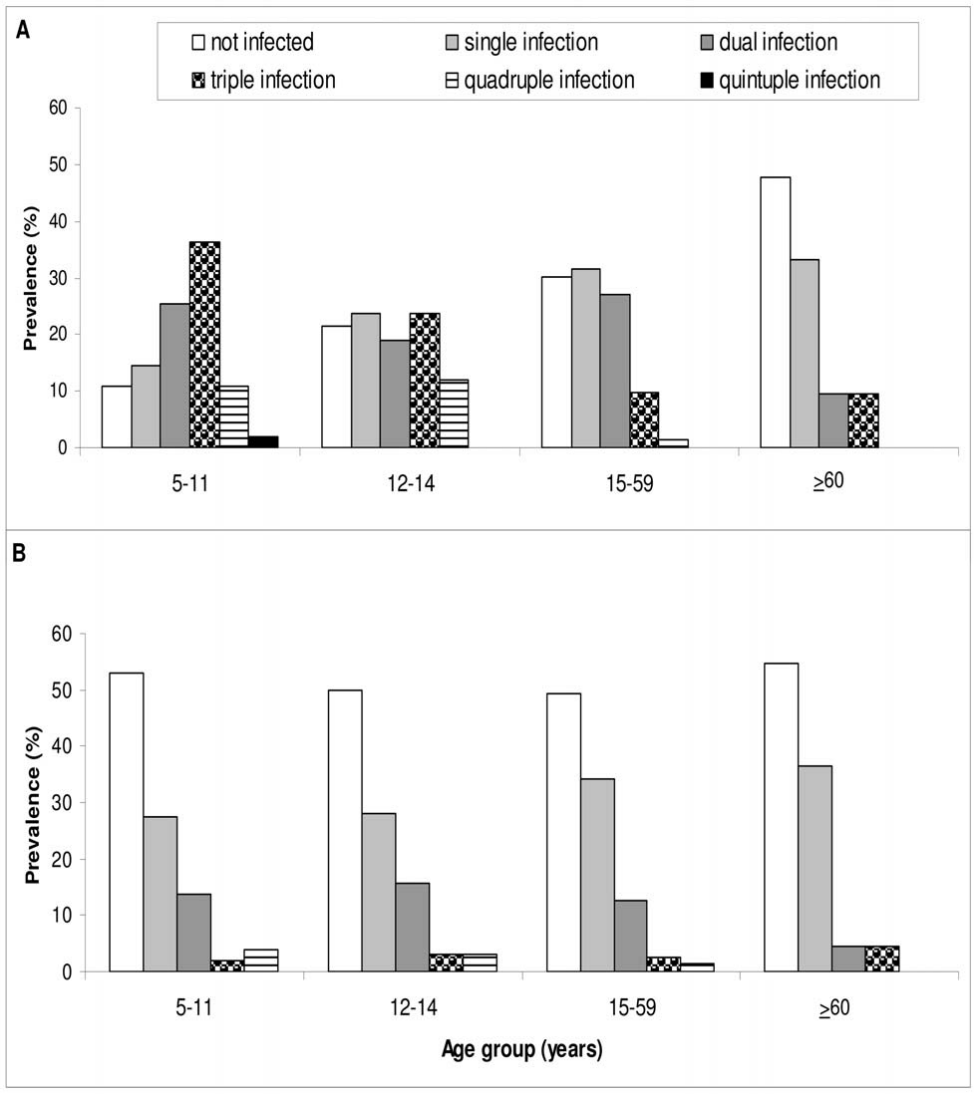
Polyparasitism in rural Bandamaji and peri-urban Dole, Zanzibar, in June/July 2008. Bar chart indicating the number of infecting helminths (polyparasitism of soil-transmitted helminths plus *S. haematobium*) in four age groups of (A) rural Bandamaji and (B) peri-urban Dole. (A) age group 5–11 years: n = 55; age group 12–14 years: n = 42; age group 15–59 years: n = 152; age-group ≥60 years: n = 21. (B) age group 5–11 years: n = 51; age group 12–14 years: n = 32; age group 15–59 years: n = 79; age-group ≥60 years: n = 22.

Figure 3 shows that patterns of polyparasitism differed according to setting and age. In rural Bandamaji, polyparasitism was highest in the youngest age group (5–11 years), with 36.4%, 10.9%, and 1.8% of the children concurrently infected with 3, 4, or even 5 helminth species, respectively. Polyparasitism decreased with age: 47.6% of the elderly (≥60 years) were free of infection. In peri-urban Dole, concomitant multiple helminth species infections were less common. Approximately half of the participants were free of any helminth infection, and concurrent infections with 3 or 4 helminths occurred in <5% of the participants of any age group. No individual was found to be parasitized with all 5 helminths concurrently.

**Figure 4:**
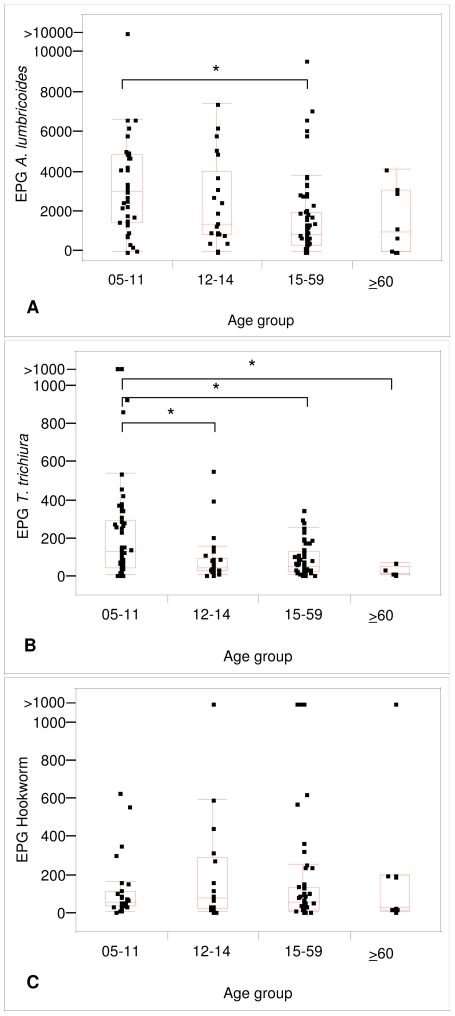
Age group specific differences in helminth infection intensities as expressed by egg excretion. Eggs per gram (EPG) values of infected individuals in different age groups of two Zanzibari communities: rural Bandamaji and peri-urban Dole. Each person's EPG was estimated as the arithmetic mean of at least two Kato-Katz thick smear readings. Differences in the median EPG of the four age groups were determined using the Kruskal-Wallis test. Pair-wise comparisons between the median EPG of two age groups were adjusted for multiple testing as suggested by Siegel and Castellan (1988) [35]. Horizontal bars are indicating the significant differences of the median EPG between two groups. Box plot: the ends of the box represent the 75^th^ and 25^th^ percentiles; the middle line represents the median; the upper whisker represents the upper quartile + 1.5*(interquartile range); the lower whisker represents the lower quartile–1.5*(interquartile range). (A): EPG values of *A. lumbricoides* infections, Kruskal-Wallis test: p<0.001; age group 5–11 years: n = 43; age group 12–14 years: n = 22; age group 15–59 years: n = 66; age-group ≥60 years: n = 8. (B) EPG values of *T. trichiura* infections, Kruskal-Wallis test: p<0.001; age group 5–11 years: n = 55; age group 12–14 years: n = 29; age group 15–59 years: n = 74; age-group ≥60 years: n = 5. (C) EPG values of hookworm infections; Kruskal-Wallis test: p = 0.789; age group 5–11 years: n = 28; age group 12–14 years: n = 18; age group 15–59 years: n = 53; age-group ≥60 years: n = 10.

Considering only arithmetic mean EPGs from infected individuals, the box plots in Figure 4 indicate that EPGs for *A. lumbricoides* were significantly higher in 5–11-year-old children than in participants aged 15–59 years (Figure 4A). EPGs for *T. trichiura* were significantly higher in the 5–11-year-old children than in the three older age groups (Figure 4B). In contrast, EPGs for hookworm showed no significant difference between age groups (Figure 4C).

### Seroprevalence of helminth infections

Positive antibody reactions against *A. lumbricoides* antigen were found in all but one tested individual (99.6%). The seroprevalence of anti-*S. haematobium* antibodies was significantly higher in Bandamaji than in Dole (46.1% *versus* 24.8%; χ^2^ = 14.1, p<0.001). The seroprevalence of *S. stercoralis* infections was 32.9% in Bandamaji and 12.8% in Dole, which revealed a highly significant difference (χ^2^ = 15.3, p<0.001). With regard to *S. haematobium*, four individuals who were found with eggs in their urine showed negative ELISA test results. Finally, 24 individuals were tested positive for *S. stercoralis* either with the KAP, or the BM, or both methods, but antibody reactions in the ELISA were judged absent. The sensitivities of the *A. lumbricoides*, *S. haematobium*, and *S. stercoralis* ELISAs were 100%, 81.8%, and 38.5%, respectively, and the specificities were 0.6%, 63.9%, and 75.0%, respectively.

### Risk factors for helminth infections and anemia, stratified by study setting

Table 2 summarizes the statistically significant (p<0.05) risk factors for helminth infections and anemia determined by multivariable logistic regression modeling, stratified by study setting. In rural Bandamaji, males had an increased risk of an *A. lumbricoides* infection (OR = 1.94, 95% CI 1.03–3.65). An incremental increase of age by 1 year reduced the risk of an *A. lumbricoides* infection (OR = 0.98, 95% CI 0.96–0.99). People consuming raw vegetables or salad were more likely to be infected with *A. lumbricoides* (OR = 2.54, 95% CI 1.27–5.10). In peri-urban Dole, no significant risk factors for an *A. lumbricoides* infection were determined.

**Table 2:** Risk factors significantly (p<0.05) associated with helminth infections and anemia in individuals from rural Bandamaji and peri-urban Dole in Zanzibar, in June/July 2008, as determined with multivariable logistic regression modeling.

Parasite	Community	Risk factor	Adjusted OR^a^	(95% CI^b^)	Wald-test P-value
***A. lumbricoides***	Bandamaji^c^	Male	1.94	(1.03, 3.65)	0.039
		Age	0.98	(0.96, 0.99)	0.004
		Eating raw vegetables or salad	2.54	(1.27, 5.10)	0.009
***T. trichiura***	Dole^d^	Age	0.97	(0.94, 1.00)	0.027
		Washing hands after defection	0.06	(0.01, 0.26)	<0.001
	Bandamaji^e^	Least poor	0.28	(0.10, 0.82)	0.020
		Age	0.96	(0.94, 0.97)	<0.001
**Hookworm**	Dole^f^	Recent travel history	5.06	(1.21, 21.06)	0.026
		Very poor	0.11	(0.02, 0.58)	0.010
		Least poor	0.12	(0.04, 0.42)	0.001
		Eating unpeeled fruits	0.28	(0.11, 0.73)	0.009
	Bandamaji^g^	Male	2.25	(1.23, 4.12)	0.008
***S. stercoralis***	Dole	Male	4.11	(1.21, 13.90)	0.023
		Washing hands after defection	0.29	(0.09, 0.96)	0.042
		Recent travel history	5.43	(1.08, 27.27)	0.040
	Bandamaji^h^	Age	0.97	(0.94, 1.00)	0.039
***S. haematobium***	Dole	Age	0.97	(0.95, 1.00)	0.022
	Bandamaji	Age	0.93	(0.90, 0.95)	<0.001
**Anemia**	Bandamaji^i^	Male	0.51	(0.27, 0.94)	0.032
		Eating raw vegetables or salad	0.45	(0.22, 0.93)	0.032

The original models included the following explanatory variables wherever expedient: the demographic variables sex and age, wealth quintiles, the risk factors washing hands with soap before eating, washing hands after defecation, washing hands with soap after defecation, consumption of raw vegetables or salad, consumption of unpeeled fruits, consumption of soil (only >5% in Bandamaji), always wearing shoes, recent travel history (only >5% in Dole), having a private toilet, sleeping under a bed net, owning a cat (only >5% in Dole), and owning a dog (only >5% in Dole). Stepwise backwards logistic regression was performed keeping only explanatory variables with P-values<0.2.

a OR  =  odds ratio.

b CI  =  confidence interval.

c Adjusted for wealth quintiles 2–5, always wearing shoes, and washing hands with soap after defecation.

d Adjusted for consumption of raw vegetables or salad, wealth quintiles 2–5, and always wearing shoes.

e Adjusted for wealth quintiles 2–4.

f Adjusted for sex, wealth quintiles 3 and 4, and washing hands with soap after defecation.

g Adjusted for age, consumption of soil, and always wearing shoes.

h Adjusted for sex.

i Adjusted for consumption of soil.

Participants from rural Bandamaji belonging to the least poor wealth quintile were at a significantly lower risk of a *T. trichiura* infection than their counterparts belonging to the most poor wealth quintile (OR = 0.28, 95% CI 0.10–0.82). In peri-urban Dole, washing hands after defecation was determined as a protective factor against a *T. trichiura* infection (OR = 0.06, 95% CI 0.01–0.26). In both study settings, for an incremental increase of age by 1 year, the risk of a *T. trichiura* infection decreased (Bandamaji: OR = 0.96, 95% CI 0.94–0.97; Dole: OR = 0.97, 95% CI 0.94–1.00).

Males from Bandamaji had an increased risk of a hookworm infection (OR = 2.25, 95% CI 1.23–4.12). In Dole, people with a recent travel history were more likely to be infected with hookworm (OR = 5.06, 95% CI 1.21–21.06). Belonging to the very poor (OR = 0.11, 95% CI 0.02–0.58) or least poor wealth quintile (OR = 0.12, 95% CI 0.04–0.42) and consumption of unpeeled fruits (OR = 0.28, 95% CI 0.11–0.73) were protective factors against a hookworm infection in Dole.

In rural Bandamaji, an incremental increase of age by 1 year reduced the risk of a *S. stercoralis* infection (OR = 0.97, 95% CI 0.94–1.00). In peri-urban Dole, males were significantly more likely to be infected with *S. stercoralis* than females (OR = 4.11, 95% CI 1.21–13.90). Moreover, a recent travel history increased the risk of a *S. stercoralis* infection in Dole (OR = 5.43, 95% CI 1.08–27.27), whereas washing hands after defecation was a protective factor (OR = 0.29, 95% CI 0.09–0.96).

In both communities an incremental increase of age by 1 year was associated with a lower risk of a *S. haematobium* infection (Bandamaji: OR = 0.93, 95% CI 0.90–0.95; Dole: OR = 0.97, 95% CI 0.95–1.00). Males from Bandamaji were less likely to be anemic than females (OR = 0.51, 95% CI 0.27–0.94), and consumption of raw vegetables or salad was a protective factor against anemia (OR = 0.45, 95% CI 0.22–0.93). In Dole, no significant risk factors for anemia were found.

### Association between helminth infections and anemia, stratified by study setting

As indicated in Table 3, an *A. lumbricoides* infection showed a significant positive association with a *T. trichiura* infection in both communities (Bandamaji: OR = 6.40, 95% CI 3.40–12.06; Dole: OR = 17.28, 95% CI 2.73–109.19). Conversely, a *T. trichiura* infection was significantly associated with an *A. lumbricoides* infection in both study settings (Bandamaji: OR = 5.38, 95% CI 2.74–10.55; Dole: OR = 20.84, 95% CI 3.92–110.75). In Dole, people with a *T. trichiura* infection where likely to harbor a concurrent *S. stercoralis* infection (OR = 5.34, 95% CI 1.39–20.56). A hookworm infection showed a significant positive association with a *T. trichiura* infection in Bandamaji (OR = 2.95, 95% CI 1.56–5.59) and with a *S. haematobium* infection in Dole (OR = 6.84; 95% CI 1.91–24.49). The multivariable regression models also showed that a *S. stercoralis* infection was positively associated with a *T. trichiura* infection (OR = 4.05, 95% CI 1.23–13.27), and that a *S. haematobium* infection was positively associated with a hookworm infection (OR = 6.89, 95% CI 1.80–26.43) in Dole. In general, heavy *S. haematobium* infections showed a strong positive association with hookworm infections (OR = 13.09; p = 0.008), and a negative association with *T. trichiura* infections (OR = 0.08; p = 0.013). Participants with an *A. lumbricoides* infection had a decreased risk of anemia in Bandamaji (OR = 0.55, 95% CI 0.31–0.98).

**Table 3:** Significant associations (p<0.05) between different helminth infections and anemia in residents from rural Bandamaji and peri-urban Dole in Zanzibar, in June/July 2008, as determined with multivariable logistic regression modeling.

Parasite	Community	Risk factor	Adjusted OR^a^	(95% CI^b^)	Wald-test P-value
***A. lumbricoides***	Dole^c^	*T. trichiura*	17.28	(2.73, 109.19)	0.002
	Bandamaji^d^	*T. trichiura*	6.40	(3.40, 12.06)	<0.001
***T. trichiura***	Dole^e^	*A. lumbricoides*	20.84	(3.92, 110.75)	<0.001
		*S. stercoralis*	5.34	(1.39, 20.56)	0.015
	Bandamaji^f^	*A. lumbricoides*	5.38	(2.74, 10.55)	<0.001
**Hookworm**	Dole^g^	*S. haematobium*	6.84	(1.91, 24.49)	0.003
	Bandamaji^h^	*T. trichiura*	2.95	(1.56, 5.59)	0.001
***S. stercoralis***	Dole^i^	*T. trichiura*	4.05	(1.23, 13.27)	0.021
***S. haematobium***	Dole^j^	Hookworm	6.89	(1.80, 26.43)	0.005
**Anemia**	Bandamaji^k^	*A. lumbricoides*	0.55	(0.31, 0.98)	0.043

The original models included the following explanatory variables wherever expedient: the demographic variables sex and age, wealth quintiles, infection with *A. lumbricoides*, *T. trichiura*, hookworm, *S. stercoralis*, and *S. haematobium*, and anemia. Stepwise backwards logistic regression was performed keeping only explanatory variables with P-values<0.2.

a OR  =  odds ratio.

b CI  =  confidence interval.

c Adjusted for sex.

d Adjusted for hookworm infection.

e Adjusted for sex, age, and wealth quintile 5.

f Adjusted for age, wealth quintile 2, and hookworm infection.

g Adjusted for age, wealth quintiles 2–5, and *A. lumbricoides* infection.

h Adjusted for sex and *S. haematobium* infection.

i Adjusted for sex.

j Adjusted for age and *T. trichiura* infection.

k Adjusted for sex and hookworm infection.

### Association between helminth infections or anemia and self-reported morbidity signs

Adjusting for demographic variables with a P-value of 0.2 or lower and stratification by community, we observed the following associations between helminth infections or anemia with self-reported morbidity signs (recall period: 2 weeks): participants from Dole with an *A. lumbricoides* (OR = 22.75, 95% CI 2.50–206.99) or *S. stercoralis* (OR = 4.47, 95% CI 1.01–19.69) infection had an increased risk of an itching body (Table 4). Participants from Bandamaji with an *A. lumbricoides* infection had a decreased risk of coughing (OR = 0.53, 95% CI 0.30–0.95), and those infected with *T. trichiura* had a decreased risk of vomiting (OR = 0.24, 95% CI 0.06–0.96). In Dole, a *T. trichiura* infection increased the risk of stomach ache (OR = 3.31, 95% CI 1.05–10.43). Participants from Bandamaji with anemia had an increased risk of an itching body (OR = 5.35, 95% CI 1.65–17.36) and an increased risk of reporting malaria (OR = 4.98, 95% CI 1.39–17.84) compared with participants without anemia. In Dole, anemia was a risk factor for fatigue (OR = 2.81, 95% CI 1.14–6.89).

**Table 4:** Self-reported morbidity signs significantly (p<0.05) associated with helminth infections and anemia among residents from rural Bandamaji and peri-urban Dole in Zanzibar, in June/July 2008, as determined with multivariable logistic regression modeling.

Reported morbidity sign	Community	Risk factor	Adjusted OR^a^	(95% CI^b^)	Wald-test P-value
Fatigue	Dole^c^	Anemia	2.81	(1.14, 6.89)	0.024
Stomach ache	Dole^d^	*T. trichiura*	3.31	(1.05, 10.43)	0.041
Vomiting	Bandamaji^e^	*T. trichiura*	0.24	(0.06, 0.96)	0.044
Cough	Bandamaji^f^	*A. lumbricoides*	0.53	(0.30, 0.95)	0.033
Itching	Dole^g^	*S. stercoralis*	4.47	(1.01, 19.69)	0.048
		*A. lumbricoides*	22.75	(2.50, 206.99)	0.006
	Bandamaji^h^	Anemia	5.35	(1.65, 17.36)	0.005
Malaria	Bandamaji^i^	Anemia	4.98	(1.39, 17.84)	0.014

The original models included the following explanatory variables: the demographic variables sex and age, and wealth quintiles, infection with *A. lumbricoides*, *T. trichiura*, hookworm, *S. stercoralis*, *S. haematobium*, and anemia. Stepwise backwards logistic regression was performed keeping only explanatory variables with P-values<0.2.

a OR  =  odds ratio.

b CI  =  confidence interval.

c Adjusted for age.

d Adjusted for sex, anemia, and *S. stercoralis*.

e Adjusted for age.

f Adjusted anemia.

g Adjusted for age, anemia, *T. trichiura*, and hookworm infection.

h Adjusted for age.

i Adjusted for age.

## Discussion

Control programs for soil-transmitted helminthiasis, schistosomiasis, and lymphatic filariasis have been implemented in Zanzibar for several years [17], [21]. The key strategy is chemotherapy-based morbidity control, using albendazole or mebendazole against soil-transmitted helminthiasis, praziquantel against schistosomiasis, and ivermectin plus albendazole against lymphatic filariasis. Importantly, the drugs used in the GPELF also show an effect against strongyloidiasis (i.e., ivermectin [13]) and against soil-transmitted helminthiasis (i.e., albendazole [20]). The helminth control programs in Zanzibar are considered successful public health interventions because of significant reductions in the prevalence and intensity of helminth infections and high levels of treatment coverage [12], [18], [21].

Analysis of our data showed, however that infections with soil-transmitted helminths and *S. haematobium* are still common, particularly in the rural setting of Bandamaji, where almost three-quarter of the participants were infected with at least one helminth species. Multiple species helminth infections affected almost half of the participants in Bandamaji, but only about one out of six individuals in the peri-urban setting of Dole. Importantly though, infection intensities were mainly low with highest EPGs observed in the youngest age group (children aged 5–11 years). Seroprevalences according to ELISA tests were 99.6% for *A. lumbricoides*, 39.2% for *S. haematobium*, and 26.4% for *S. stercoralis*, but the test specificities were low. More than half of the participants were anemic and, interestingly, the overall prevalence of anemia in peri-urban Dole was significantly higher than in rural Bandamaji (64.7% *versus* 50.9%).

Two important limitations of our study are that we did not adhere to a strict randomization procedure for enrollment, and that the number of fully complying individuals was rather low. According to estimates for the year 2007, the total population in Bandamaji and Dole were 1,118 and 2,876, respectively. Hence, our final study cohort consisted of approximately 30% of the population of Bandamaji and 13% in Dole. With regard to the number of fully complying individuals, one should bear in mind that we aimed at collecting three consecutive stool samples per person, employing a suite of diagnostic methods, and that we worked with all age groups of two communities. Repeated stool sampling reduced the study compliance from 79% to 69% for the submission of the first to the second stool sample, and to a level of 48% for submission of all three stool samples. The overall compliance rate is different to the one we reached with repeated stool sampling among school children in two schools in Zanzibar (85%) [30]. School children are, however, readily accessible and the education system provides a convenient platform for deworming campaigns, whereas in the community, research teams and program officers depend on the will and stamina of the individuals not to forget submitting a filled stool container every morning without a daily reminder. The low compliance to the questionnaire survey and concomitant provision of a blood and urine sample is likely a result of the time consuming procedure, competing with other daily activities.

Since treatment coverage by the GPELF implemented from 2001–2006 in both study sites was equally high (mean in Bandamaji: 81.9%, mean in Dole: 83.0%) and school-based deworming reached coverage levels of 75% of the at-risk population in Zanzibar in 2006, there must be other local risk factors abetting different levels of helminth infections and anemia. The distinctive age-dependent patterns of *A. lumbricoides*, *T. trichiura*, and *S. haematobium* infection prevalence and intensity in our study population are consistent with the literature [39]. All other identified risk factors in our study showed setting-specific idiosyncrasies. For example, *S. stercoralis* infections showed no clear age-profile, but males were at a several-fold higher risk of an infection than females in Dole, similar to the observed gender difference for hookworm infection in Bandamaji. It should be noted that reports on the prevalence and age-profile of *S. stercoralis* infections are rare, often with conflicting results. Whilst studies from Côte d'Ivoire and China reported a higher prevalence of strongyloidiasis in adults [40], [41], in the Peruvian Amazon and in aboriginal communities in Australia mostly children were affected [42], [43]. In line with findings from Jamaica, our data suggest that an infection with *S. stercoralis* is independent of age [44]. The observation of a higher *S. stercoralis* prevalence among males confirms results obtained elsewhere [42], [45], but is in contrast to recent findings from Côte d'Ivoire [46]. The higher risk of both hookworm and *S. stercoralis* infections in males is likely a result of genetic and immunologic determinants, as well as of gender-specific risk behavior. Moreover, and depending on the study setting, several behavioral factors were identified that showed significant associations with helminth infections and anemia: consumption of raw vegetable or salad was a risk factor for an infection with *A. lumbricoides*, whereas washing hands after defecation and socio-economic status (least poor wealth quintile) were significant protective factors against a *T. trichiura* infection. People washing hands after defection were also less likely to be infected with *S. stercoralis*. A recent travel history was associated with a higher risk of both hookworm and *S. stercoralis* infection. Males and the participants consuming raw vegetables or salad were at a lower risk of anemia. In addition to age, sex, socio-economic status, and personal behavior, we believe that there are setting-specific sanitary and environmental risk factors that might abet helminth infections. For example, in District North A, 46% of all households had no toilet facilities in 2004/2005, whereas in District West only 8% of households had no access to toilet facilities [47]. Hence, the environmental contamination with helminth eggs or larvae is likely to be higher in District North A, and people are at a higher risk of soil-transmitted helminth infections. Moreover, the survival and longevity of helminth eggs or larvae in the natural environment depend on soil type and vegetation, which has previously been discussed for Zanzibar and other African settings [11], [48], [49].

The positive associations between (i) *A. lumbricoides* and *T. trichiura*, (ii) hookworm and *T. trichiura*, (iii) *S. stercoralis* and *T. trichiura*, and (iv) hookworm and *S. haematobium* observed in the current study in Unguja are in line with previous investigations on helminth associations carried out in Unguja and Pemba [10], [12]. Interestingly, the adjusted OR indicating an association of hookworm and *S. haematobium* infection increased from 4.3 to 13.1 if children harbored heavy *S. haematobium* infections. This observation is similar to findings from Côte d'Ivoire and Brazil where children with increasing infection intensity of *S. mansoni* were also more likely to be concurrently infected with hookworm [50], [51]. Hookworm and *Schistosoma* spp. infections are leading causes of anemia that can result in growth retardation and cognitive impairment of children [52], [53], [54]. Hence, if hookworm infections are associated with or even exacerbate with a concurrent schistosome infection, the risk of chronic anemia and related morbidity is likely to be elevated in co-endemic settings. To control morbidity due to multiple helminth infections, triple co-administration of albendazole, praziquantel, and ivermectin might be an effective strategy. Triple co-administration of the respective drugs has been shown to be safe in co-endemic settings in Zanzibar, where multiple rounds of treatment had been implemented in the past [17]. Since demographic factors, personal risk behavior, and socio-economic status shape the profile of helminth infections, poverty alleviation strategies complemented with health education and improved access to clean water and adequate sanitation, in addition to regular deworming, can help to decrease the burden of helminth infections in Africa and elsewhere in the developing world [3], [55].

The high prevalence rates of anemia observed in both settings of our study indicate that anemia is still a major public health problem in Zanzibar, which begins early in life [56]. Participants from the rural setting presenting with anemia were more likely to report a malaria infection within the last two weeks. The association between malaria and anemia is well documented [57]. Interestingly, people infected with one or several helminth species concurrently were not at a higher risk of anemia compared with non-infected individuals. Our results are therefore in contrast to a study carried out in the Philippines, where individuals with multiple species helminth infections of light intensity were at an elevated risk of anemia [6]. In the current study, participants from Bandamaji infected with *A. lumbricoides* were at a lower risk of anemia and cough, and those infected with *T. trichiura* were less likely to report vomiting within the past two weeks. These findings might point to a potent immuno-modulation by helminths resulting in disease protective immune responses [58]. Since all findings were setting-specific it is, however, also conceivable that the apparent associations were due to local social or environmental determinants, as suggested elsewhere [59]. We were surprised to find a higher prevalence of anemia in the peri-urban community than in rural Bandamaji. Our results might suggest that anemia was driven not only by malaria but also by nutritional factors, and perhaps ethnicity, rather than by (multi)helminth infections [60], [61].

Sero-prevalences of *A. lumbricoides*, *S. stercoralis*, and *S. haematobium*, as determined by ELISA, were several-fold higher than the prevalences found with standard direct diagnostic methods for eggs and larvae, but it must be emphasized that the specificities of all performed ELISAs were low. Likely reasons for these observations are as follows. First, whilst the prevalences and intensity of helminth infections have significantly decreased as a result of large-scale deworming programs in Zanzibar [21], antibodies from past infections can persist for a prolonged period of time after successful treatment, and hence be detected with ELISA [62], [63]. Second, it is known that unspecific cross-reactions (e.g., from antibodies against antigen from *Ascaris* or filarial worms) can occur [64], [65]. Third, widely used parasitological techniques such as the K-K thick smear lack sensitivity for detecting low-intensity helminth infections [30], [66]. Indirect diagnostic tools such as ELISA might be more sensitive [67]–[69].

While there are many explanations for higher helminth seroprevalences determined with ELISA, it is unclear why the ELISAs failed to detect four *S. haematobium* and 24 *S. stercoralis* infections diagnosed by microscopy. Most study participants, however, had either a very low antibody response to all performed ELISAs or the measured OD was marginal below the cut-off level, suggesting that the thresholds proposed in the manufacturer's manual need careful revision, at least for our study setting. Summarizing, our data confirm that people living in areas highly endemic for helminthiases are immunologically activated as a result of previous infections [70]. Since helminths are masters in modulating host immunity they are likely impacting on co-infections, allergy, and immunizations [58]. Therefore, it will be important to incorporate gained knowledge on the epidemiology of immunological markers in future public health decisions.

Our study indicates that, despite considerable progress made in the control of helminthiases in Zanzibar [12], [18], [21], the “worm-problem” and anemia in Zanzibar remains a formidable challenge and cannot be overcome by preventive chemotherapy alone [71]. It should be noted that the GPELF, which regularly deployed albendazole plus ivermectin and likely had a beneficial effect on soil-transmitted helminthiasis, was terminated in 2006. The patterns of helminth infections and anemia in rural and peri-urban communities and the identified risk factors emphasize that the pressure of helminth transmission in Zanzibar is still pervasive and that additional control measures are needed to consolidate progress made to date with preventive chemotherapy. With new discussions exploring options for further reduction of helminthiases in Zanzibar and elsewhere, including shifting the focus from morbidity control to transmission control, there is a need for integrated control programs, acting beyond preventive chemotherapy [55], [72], [73]. Indeed, greater steps should be taken to enforce health education, and action is needed to improve access to clean water and adequate sanitation (e.g., by community-led total sanitation). These measures will also result in an enhanced socio-economic status of people, and hence alleviate poverty, which is the key factor for the control and ultimate elimination of helminthiases.

## Supporting Information

Alternative Language Abstract S1 Translation of the abstract into German by SK.
(0.03 MB DOC)

Checklist S1 STROBE checklist.
(1.37 MB PDF)
