# Supplementary material for: Patterns and Risk Factors of Helminthiasis and Anemia in a Rural and a Peri-urban Community in Zanzibar, in the Context of Helminth Control Programs
Source: PLoS Negl Trop Dis. 2010 May 11;4(5):e681. doi: 10.1371/journal.pntd.0000681 (PMC2867941; doi:10.1371/journal.pntd.0000681)
Supplement: Alternative Language Abstract S1 — Translation of the abstract into German by SK. (0.03 MB DOC) [file pntd.0000681.s001.doc]

**Patterns and Risk Factors of Helminthiasis and Anemia in a Rural and a Peri-Urban Community in Zanzibar, in the Context of Helminth Control Programs:**

**Muster und Risikofaktoren von Wurminfektionen in einer ländlichen und einer vorstädtischen Bevölkerung in Sansibar, im Kontext von Wurmkontrollprogrammen**

**Zusammenfassung**

***Hintergrund:*** Die Kontrolle von Wurminfektionen und die Vorbeugung von Blutarmut sind wichtig zur Verbesserung des Gesundheitswesens in Entwicklungsländern. Es war unser Ziel die Muster und Risikofaktoren von Wurminfektionen und Blutarmut in einer ländlichen und in einer vorstädtischen Bevölkerung in Sansibar, Tansania, im Kontext von nationalen Wurmkontrollprogrammen zu beschreiben.

***Methoden/wichtigste Ergebnisse:*** Wir führten eine Querschnittsstudie mit 454 Personen aus zwei Dorfschaften durch und untersuchten mindestens zwei Stuhlproben mit unterschiedlichen Methoden auf parasitäre Würmer (Rundwurm, Hakenwurm, Zwergfadenwurm und Peitschenwurm) und eine Urinprobe auf den Erreger der Blasenbilharziose (*Schistosoma haematobium*). Kapillares Blut aus dem Finger wurde benutzt um Blutarmut und Antikörperreaktionen gegen den Rundwurm, Zwergfadenwurm und die Blasenbilharziose mit immunologischen Tests festzustellen. Bei rund drei Vierteln der ländlichen Dorfschaft (73.7%) und bei knapp der Hälfte der vorstädtischen Bevölkerung (48.9%) diagnostizierten wir Wurminfektionen. Die meisten Infektionen waren leicht und Kinder im Schulalter waren am stärksten infiziert. Infektionen mit mehreren Wurmarten gleichzeitig waren unabhängig vom Alter in der ländlichen Bevölkerung häufiger. Mehr als die Hälfte der Studienteilnehmer und besonders die vorstädtische Bevölkerung litt unter Blutarmut. Risikofaktoren für Wurminfektionen waren Alter, Geschlecht, der Verzehr von rohem Gemüse oder Salat, Reisen ausserhalb der Gemeinde und der sozioökonomische Status.

***Schlussfolgerung/Bedeutung:*** Seit mehreren Jahren führt Sansibar ein nationales Wurmkontrollprogramm durch, welches vorwiegend auf medikamentöser Behandlung zur Vorbeugung von Erkrankungen beruht. Wurminfektionen und Blutarmut sind allerdings immer noch häufig. Die Intensitäten der Wurminfektionen sind hingegen niedrig. Aus diesem Grund schlagen wir vor, dass die Behandlung fortgeführt wird. Um eine nachhaltigere Wirkung zu erzielen, sollten aber im Einklang mit weiteren Armutsbekämpfungsmaßnahmen auch der Zugang zu sauberem Trinkwasser, adäquaten sanitären Einrichtungen und Gesundheitserziehung verbessert werden.

***Übersetzung:*** Stefanie Knopp
